# Melatonin-treated bone marrow mesenchymal stem cell-derived exosomes reverse liver fibrosis induced by CCl4 in male wistar albino rats

**DOI:** 10.1038/s41598-026-58433-x

**Published:** 2026-06-21

**Authors:** Naglaa W. Abdelbaky, Ahmed Nabil, Osama M. Ahmed, Mohamed I. Zanaty

**Affiliations:** 1 Biotechnology and Life Sciences Department, Faculty of Postgraduate Studies for Advanced Sciences, Beni-Suef University, Beni-Suef, 62511 Egypt; 2 Zoology Department, Faculty of Science, Beni-Suef University, Beni-Suef, 62511 Egypt

**Keywords:** Fibrosis, Mesenchymal Stem Cells, Exosomes, Melatonin, Inflammatory Cytokines, Apoptotic Markers, Fibrotic Markers, Biochemistry, Cell biology, Diseases, Medical research, Molecular biology

## Abstract

Liver fibrosis is defined as the substitution of hepatocytes with extracellular matrix (ECM) proteins, accompanied by abnormalities in matrix remodelling. We aimed to assess the efficacy of melatonin-pretreated bone marrow mesenchymal stem cell-derived exosomes (MT/Exos) in mitigating liver fibrosis. MT/Exos were isolated from their parent cells and characterised by measuring protein content, particle size, and cluster of differentiation (CD) markers. In vivo studies (MT/Exos (100 µg/rat) via the caudal vein twice weekly for 4 weeks) include assessment of liver biomarkers, oxidative stress markers, inflammatory cytokines, apoptotic markers, fibrotic cytokines, and histopathological changes. MT/Exos with 1 mg/ml protein showed double-membrane vesicles sized 50–100 nm, and expressed CD63 and CD81 markers. Treated rats showed restoration of albumin levels, significant improvements in liver enzymes, notable elevations in SOD activity and Nrf2 levels, and a marked reduction in MDA levels. TNF-α, IL-17, NF-κB-p50, and p65 levels were attenuated, and IL-10 levels increased significantly. p53, Bax, caspase-3, TGF-β, SMAD3, collagen I, and miRNA 196 also showed a substantial decrease in treated rats (P < 0.05). Histopathological examination confirmed a marked reduction of collagen deposition and inflammatory infiltration. MT/Exos were successfully isolated and characterized. The MT/Exos-treated group showed improvements in all liver function parameters, with increased levels of antioxidant and anti-inflammatory markers. MT/Exos reduced apoptotic and fibrotic modulators and improved histopathological features. These findings confirm the synergistic effect of melatonin preconditioning in improving the therapeutic potential of MSC-derived exosomes as a novel anti-fibrotic agent. Overall, MT/Exos may offer a promising therapeutic approach for liver fibrosis with potential clinical relevance pending further studies.

**Supplementary Information:**

The online version contains supplementary material available at 10.1038/s41598-026-58433-x.

## Introduction

Liver Fibrosis is a pathological condition that represents a key risk factor for many liver diseases, including cirrhosis and liver cancers^1^. Underlying etiologies include alcohol-induced, autoimmune diseases, and metabolic disorders^2^. Liver fibrosis is characterised by the replacement of the normal architecture of liver tissues by dysfunctional fibrotic tissues consisting of collagen I/III ECM proteins^3^. During hepatic fibrogenesis, hepatic stellate cells (HSCs) represent the principal source of ECM deposition; therefore, they play a critical role in preserving liver function and structural homeostasis. HSCs are located in the Disse space positioned between hepatocytes and liver sinusoids in a quiescent state. Pathologically, in response to hepatic injury or inflammation, pro-inflammatory and profibrotic hepatokines, such as transforming growth factor beta (TGF-β), are produced by Kupffer cells. These modulators induce the transformation of HSCs from quiescent inactive cells into myofibroblast-like cells^4^. Upon activation, HSCs produce large amounts of collagen and other ECM proteins, as well as TGF-β, which further promote continuous activation and proliferation of HSCs^5^. Deposition and accumulation of collagen form a network of interconnecting fibrous barriers that distort the standard hepatic architecture and impair the central metabolic and detoxification functions^6^. Indeed, HSC activation has a crucial role in the fibrogenic cascade in the liver; thus, inducing HSC senescence could be an effective mechanism to reverse the development of liver fibrosis^7^. While partial reversal of fibrosis has been documented following removal of the causative insult, no approved therapy currently achieves consistent antifibrotic efficacy. MSC transplantation could target HSC inhibition and trigger reversal of the fibrotic process, thereby alleviating hepatic fibrosis^8^. Stem cells possess unique self-renewal and differentiation capabilities, as well as the ability to give rise to many progenitor cell types, including liver progenitor cells. Among different stem cell types, MSCs exhibited promising efficacy in regenerative medicine. MSCs are multipotent progenitor cells that show potent therapeutic efficacy for tissue repair and tissue engineering in various diseases^9,10^. MSCs transplantation has revealed a positive impact in the amelioration of liver fibrosis^11,12^. However, clinical trials reported the therapeutic capability of MSCs in hepatic diseases^13,14^. Cellular rejection and tumor formation remain critical concerns^15,16^. Recent evidence suggests that MSCs’ therapeutic effects are primarily exerted through paracrine signalling, mainly via the release of extracellular vesicles (EVs)^17^. Moreover, MSC-derived EVs have emerged as a cell-free therapy to avoid the drawbacks of MSC transplantation^18,19^. Exosomes are an EVs described as a molecular bioprint of their parent cells. MSC-derived Exos have many advantages over their corresponding cells; they are smaller in size (30–100 nm), therefore easier to produce and store, and have low immunogenicity, so there is no risk of tumor formation^20,21^. Exosomes act as carriers for a variety of biomolecules, such as lipids, nucleic acids, and proteins; their cargoes varies according to the origin and biological state of exosomes^22^. Exosomes’ interactions with recipient cells include membrane fusion, phagocytosis, receptor-mediated endocytosis, and micropinocytosis^23^. It has promising potential to enhance tissue repair, reduce organ inflammation and oxidative stress, and inhibit fibrosis^24^. The therapeutic potential of MSC-derived exosomes has been demonstrated in many pathological conditions^20,25^. The yield of exosomes is limited by their parent cells, and their efficacy needs to be enhanced. To overcome these issues, preconditioned methods can enrich MSCs’ biological functions and enhance their paracrine properties, thereby improving the therapeutic impact of their derived EVs^26,27^. Recent studies reported that melatonin (MT) preconditioning modulates MSCs by enhancing their survival, proliferation, and paracrine activity under pathological conditions^28^. Melatonin, a pineal gland hormone, has antioxidant and anti-inflammatory properties. A previous study suggested that melatonin could improve the efficacy of MSCs-derived exosomes in the treatment of renal injury of ischemia-reperfusion^29^. To our knowledge, no previous study has investigated whether melatonin preconditioning of MSCs can enhance the therapeutic efficacy of their derived exosomes against liver fibrosis. Therefore, we aimed to investigate the therapeutic efficacy of exosomes derived from MT-pretreated BM-MSCs in reversing CCl4-induced liver fibrosis in Wistar albino rats.

## Methods

### Preparation, isolation, and characterisation of bone marrow MSCs and their derived exosomes

#### Melatonin-pretreated bone marrow MSCs culturing and characterization

A three-week-old albino rat (40–50 g) was used for collecting bone marrow. Rats were obtained from the Helwan farm unit of the Laboratory Animal VACSERA, Egypt. Rats were anesthetized according to their body weight by intraperitoneal injection of ketamine (75 mg/kg) and xylazine (10 mg/kg), followed by cervical dislocation for euthanasia. After cervical dislocation, the rat’s tibia and femur were separated according to the isolation protocol of Sauter et al.^30^. In brief, the harvested bone marrow was transferred into a low-glucose DMEM T-25 flask, with 10% FBS and 1% antibiotic. The incubation condition involved a 5% CO_2_ cell culture incubator at 37 °C and 95% humidity. The flask medium was replaced every four days. Cells must undergo subculturing when their density reaches 70–80%. The cells were detached using 0.25% trypsin–EDTA for 3 min at 37 °C. Then, the enzymatic activity of trypsin was inhibited with complete medium. The suspension was centrifuged, and the precipitated pellet was transferred to a new flask containing complete medium and incubated under the same conditions as in the previous passage. Melatonin -pretreated MSCs were cultured using the same protocol, with the addition of 50 µM melatonin (99%, Thermo Fisher Scientific Company) to the medium^31^. Passage four from treated and untreated MSCs was used to isolate extracellular vesicles, mainly exosomes. Morphological characterization of cultured MSCs was performed using an inverted microscope, and identification of CD markers was conducted by flow cytometry. MSCs were washed, resuspended, and incubated in phosphate buffer saline supplemented with 3% FBS and fluorescein isothiocyanate-conjugated monoclonal antibodies against CD73, CD90, CD105, and CD34, and a forward-scattering analytical method was used for sample analysis^32^.

#### MSCs-derived exosomes isolation and characterization

MT-treated and untreated MSCs at passage IV were used to isolate exosomes. The cells were cultivated in an FBS-free culture medium containing 0.5% bovine serum albumin for 24 h. After incubation, conditioned media were harvested and first centrifuged at 2000 xg for 20 min. The centrifuged media were separated into cells and debris-containing pellet and supernatant containing EVs and exosomes. This supernatant was subjected to ultracentrifugation at 100,000xg for 32 min at 4 °C using a fixed-angle rotor on a Sorvall MTX 150 mini-ultracentrifuge (Thermo Fisher Scientific). The purified pellet containing exosomes was resuspended in 100 µL PBS, and its protein content was assessed using the bicinchoninic acid (BCA) protein assay kit (Novagen) according to the protocol of Smith et al.^33^. The vials containing exosomes were stored at -80 °C for subsequent characterization and in vivo administration to rats. A high-resolution transmission Electron Microscope (HR-TEM) (HR-TEM; JEOL JEM-2100, Tokyo, Japan) was used to characterize exosome morphology; exosomes were loaded onto copper grids and negatively stained with phosphotungstic acid, then analyzed by TEM^34^.

### In vivo study

#### Experimental animals

Forty male Wistar albino rats were used in our study; they were 8–10 weeks old and weighed 130–150 g each. Rats were obtained from the Helwan farm unit of the Laboratory Animal VACSERA, Egypt. The in vivo experiment was maintained following the guidelines of the Animal Resources Institute for Laboratory Animal Care and Use, and the Animal Care Committee of Beni-Suef University (PSAS-BSU-HAREC, protocol number 024–058). All rats were housed up to five per cage following 12 a 12-hour light/dark cycle. They had free access to food and water at an ambient temperature of 20–25 °C and relative humidity of about 40–60%. All procedures comply with ethical guidelines for animal testing and research. After acclimatization, rats were divided into normal control rats (*n* = 9) and fibrosis model rats (*n* = 31).

#### Induction of fibrosis

31 rats received subcutaneous injection of 1 ml/kg of CCl4 solution twice a week for 9 consecutive weeks^35^. CCl4 was diluted by 50% (v/v) with olive oil^36^. CCl₄ administration was discontinued after the established liver fibrosis model, and before the 4-week treatment period. Only 2 rats died during the 9 weeks of induction. Moreover, two rats were sacrificed at the end of the fibrosis induction period; their serum and liver tissues were analyzed biochemically and histopathologically to confirm the successful induction of hepatic fibrosis before the initiation of treatment, and their data were identically matched with that of the control positive group and were not involved in the study design. The treatment protocol started at week 10, after completion of the 9-week CCl₄ fibrosis induction period and confirmation of hepatic fibrosis.

#### Experimental design

**Group I** (*n* = 9) served as the normal control and received placebo treatment throughout the four-week experimental period.

**Group II** (CCl4 rats) served as the positive control (*n* = 9). CCl4-intoxicated rats (1 ml/kg of CCl4 in 50% (v/v) olive oil) did not receive any treatment throughout the four-week treatment period. **Group III** (CCl4 + Exos), (*n* = 9): rats were administered Exos (100 µg/rat) via the caudal vein twice weekly for 4 weeks during the treatment protocol^37^.

**Group IV** (CCl4 + MT/Exos) n (*n* = 9): rats were administered MT/Exos (100 µg/rat) via the caudal vein twice weekly for 4 weeks during the treatment protocol.

### Sample collection and tissue preparation

24 h after the last administered dose, blood samples were drawn from the median plexus of the orbit under deep anaesthesia induced by intraperitoneal injection of ketamine (80 mg/kg) and xylazine (10 mg/kg). After that, rats were euthanised by cervical dislocation, and all procedures were performed according to ARRIVE guidelines. Blood specimens were centrifuged at 4000 rpm, 4 °C for 20 min. The clear serum was separated and used to assess liver function. Liver tissues were surgically excised and rinsed several times with physiological saline to remove blood cells and any attached debris. Each liver was divided into three segments. The first portion was immediately flash-frozen at − 80 °C for subsequent Western blot and molecular investigations. The second part (0.5 g) was added to 5 mL of ice-cold saline and homogenized using a tissue homogenizer. Then, the samples were centrifuged at 3000 rpm for 20 min at 4 °C to remove the cell debris. Tissue homogenates were well preserved for evaluating hepatic oxidative stress modulators and estimating IL-17, TNF-α, and IL-10 values. The remaining hepatic tissue was rinsed again with cold saline, then immersed in 10% buffered formaldehyde for fixation prior to histological analysis.

### Liver function markers determination

Albumin and liver aminotransferase levels were assessed in the serum samples, free of any homolysis. Bio-diagnostic commercial albumin kits, Alanine Aminotransferase (ALT), and Aspartate Aminotransferase were used (^®^ kits Cat# AB 1010, AL 10 31, and AS 10 61 Cairo, Egypt) by a biochemical analyzer Rebonik (AT2450317RBK).

### Evaluation of oxidative stress biomarkers in the liver homogenate

The contents of Malondialdehyde (MDA) and superoxide dismutase (SOD) in hepatic tissues was determined colorimetrically using a biochemical analyzer Rebonik (AT2450317RBK), and commercial kits: MDA (cat# MD2529) and SOD (cat# SD2521), purchased from Bio Diagnostic Company, Egypt.

### Assessment of hepatic inflammatory cytokines

Inflammatory markers include proinflammatory Interleukin-17(IL-17) and tumor necrosis factor (TNFα), and anti-inflammatory interleukin-10 (IL-10). Protein concentrations of these markers were determined in liver homogenate using commercially obtainable Enzyme-Linked Immunosorbent Assay (ELISA) kits; TNFα Cat. No. 438,204, IL-17 Cat. No: ER0035 and IL-10 Cat. No SEA056Ra.

### Assessment of protein values of apoptotic, profibrotic modulators, Nrf2, and NF-KB 65 and 50 subunits using western blotting

Liver tissue samples were lysed under cold conditions, and three samples from each group were used in western blotting investigations. Firstly, total protein content in each sample was extracted according to the protocol of^38^ using the Extraction Kit (Bio-Rad Inc). The concentration of purified protein was quantified via Bradford Protein Assay Kit (SK3041). The next step is SDS–PAGE electrophoresis; equal amounts of protein were separated using the TGX Stain-Free™ FastCast™ Acrylamide Kit. Proteins were then transferred to polyvinylidene fluoride membranes and blocked in Tris-buffered saline, pH 7.5, for one hour at room temperature. The membrane was subsequently incubated overnight at 4 °C with diluted primary antibodies according to the manufacturer’s instructions against p53, Bax, cleaved caspase-3, TGF-β, SMAD3, collagen I, Nrf2, and NF-κB (p65 and p50). The blot was then washed with tris-buffered saline with Tween 20 buffer for 5 min. After washing, horseradish peroxidase (HRP)-conjugated secondary antibodies were incubated with the membranes for 1 h at room temp. Chemiluminescent signals were detected using the Bio-Rad Clarity™ Western ECL substrate and visualized with a CCD camera-based ChemiDoc MP imaging system. The intensity of target protein Bands was quantified relative to β-actin, a control, by image analysis software. All antibodies were purchased from Santa Cruz Biotechnology, Inc.

### Molecular investigation of miRNA 196

Total RNA was purified from serum samples using the Direct-zol™ RNA Miniprep Plus kit (Zymo Research Corp., USA; Cat. #R2072). RNA concentration and purity have been assessed using a Beckman dual-spectrophotometer (USA). The SuperScript IV One-Step RT-PCR kit (Thermo Fisher Scientific, USA, Cat# 12594100) was used for reverse transcription and amplification according to the manufacturer’s instructions using SuperScript™ IV RT Step One (Applied Biosystems, Foster City, USA). The primer sequences used are listed in Table 1. Quantitative data were reported as cycle threshold (Ct) values. The Ct values obtained for miR-196 were normalized to those of the corresponding housekeeping gene (U6). The relative expression levels were calculated using the 2 − ^ΔΔ^Ct method.

**Table 1:** The primer sequence of miRNA 196 and the housekeeping gene (U6).

	Forward sequence	Reverse sequence
*miR-196*	ATCCTTCCTAGTCCAGCCTGAG	ACCTGGCGGCACTCCTTA
*U6*	ATGACGTCTGCCTTGGAGAAC	TCAGTGTGCTACGGAGTTCAG

### Histopathological testing

The liver samples used for histopathological analyses were cut into approximately 0.1 g. Samples were immediately fixed in 10% neutral buffered paraformaldehyde for 24 h at room temperature. Dehydration of the samples was achieved by a graded ethanol dehydration method. The samples were then embedded in paraffin wax and sectioned at a thickness of four µm. The well-prepared tissue slides were subsequently stained using hematoxylin and eosin H&E and Masson’s trichrome stains. A qualified pathologist examined the stained slides under a light microscope for histological assessment of hepatic architecture, inflammatory changes, and fibrotic alterations^39^.

### Statistical analysis

All statistical analyses were performed using IBM SPSS Statistics version 22.0 (IBM Corp., Armonk, NY, USA). Results are presented as mean ± SEM. One-way ANOVA accompanied by Duncan’s post hoc test was employed to evaluate group differences, with *p* < 0.05 denoting statistical significance.

## Results

### Bone marrow MSCs characterization

**Fig. 1:**
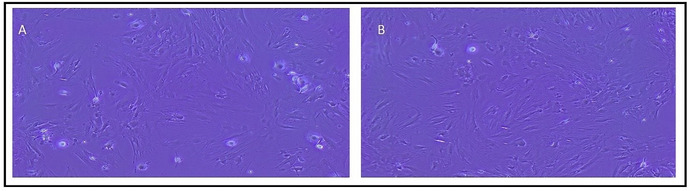
Inverted microscope morphological characterization of (**A**) MSCs and (**B**) MT/MSCs.

**Fig. 2:**
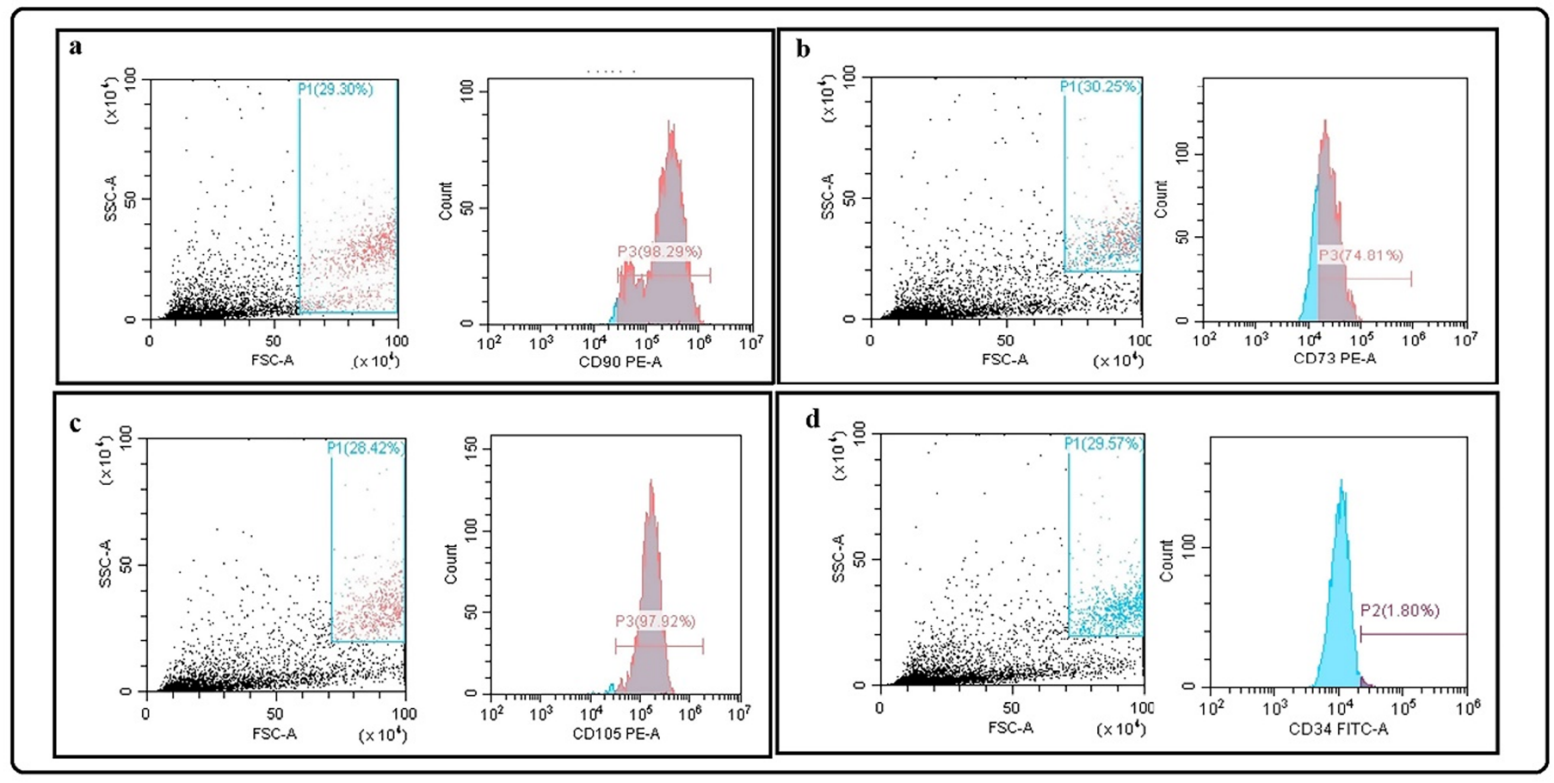
Flow cytometry of MSCs; (**a**) symbol represents CD 90 by 98.29%, (**b**) represents positive expression of CD 73 by 74.01%, (**c**) represents positive expression of CD 105 by 97.92% and d represents negative expression of CD 34 by 1.80%.

**Fig. 3:**
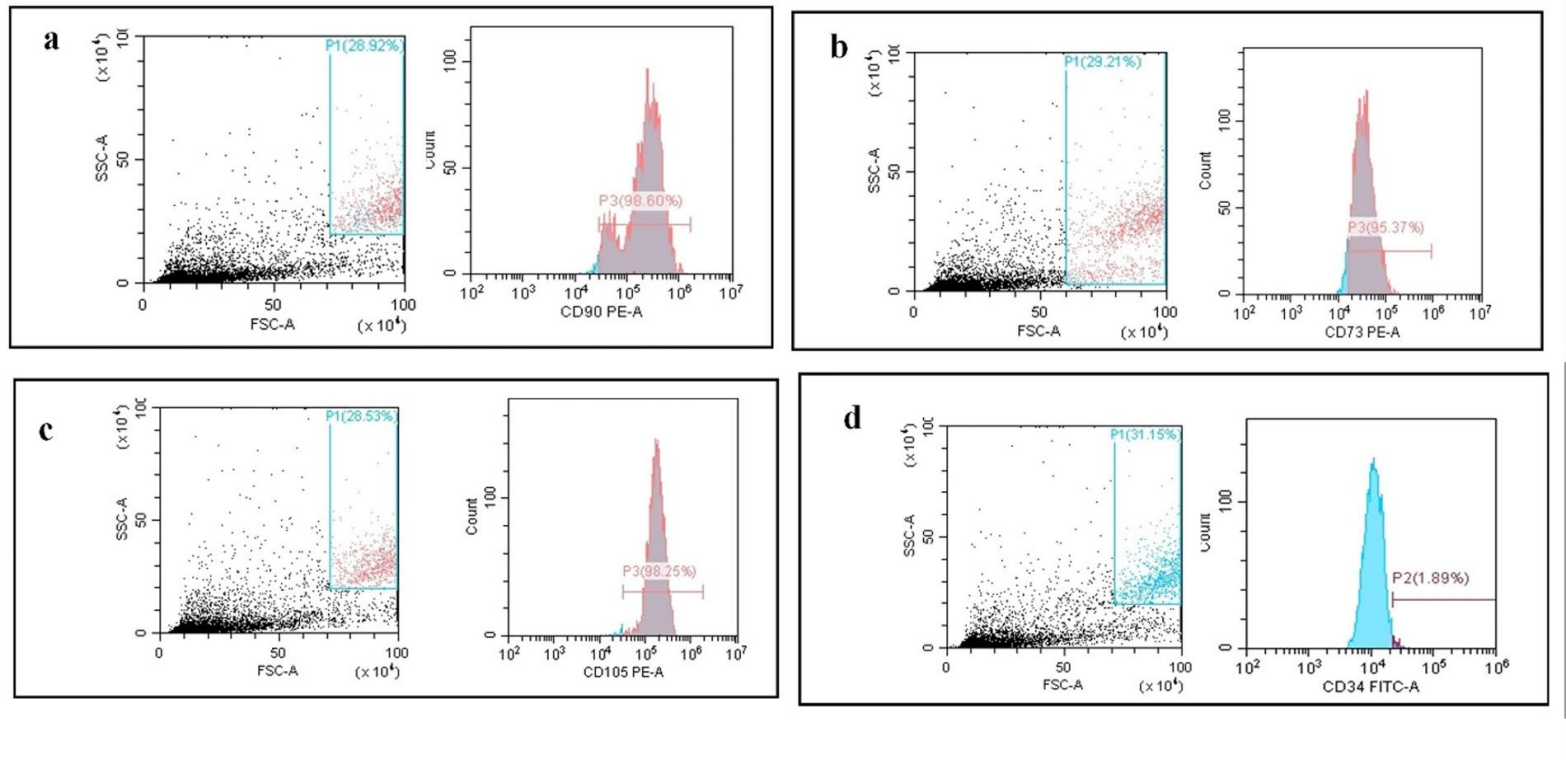
Flow cytometry of MT/MSCs; (**a**) symbol represents CD 90 by 98.6%, (**b**) represents positive expression of CD 73 by 95.73%, (**c**) represents positive expression of CD 105 by 98.25%, and d represents negative expression of CD 34 by 1.89%.

Bone marrow MSCs cultivated on melatonin-treated and untreated media were examined by an inverted microscope and exhibited ideal morphological traits. As shown in (Fig. 1A), cells were adherent and spindle-shaped, displaying typical spindle and fibroblast-like morphologies. Flow cytometry results indicated that purified MSCs expressed positive levels of the tested CD markers (CD73, CD90, and CD105) and were negative for CD34. Figure 2: Untreated MSCs expressed 74.81% of CD73, 98.29% of CD90, 97.94% of CD105, and showed 1.8% negative expression for CD34. Figure 3: MT/MSCs showed positive marker expression of 95.73% for CD73, 98.6% for CD90, 98.25% for CD105, and 1.89% negative for CD34.

### Quantification and characterization of MT/MSCs‑derived exosomes

**Fig. 4:**
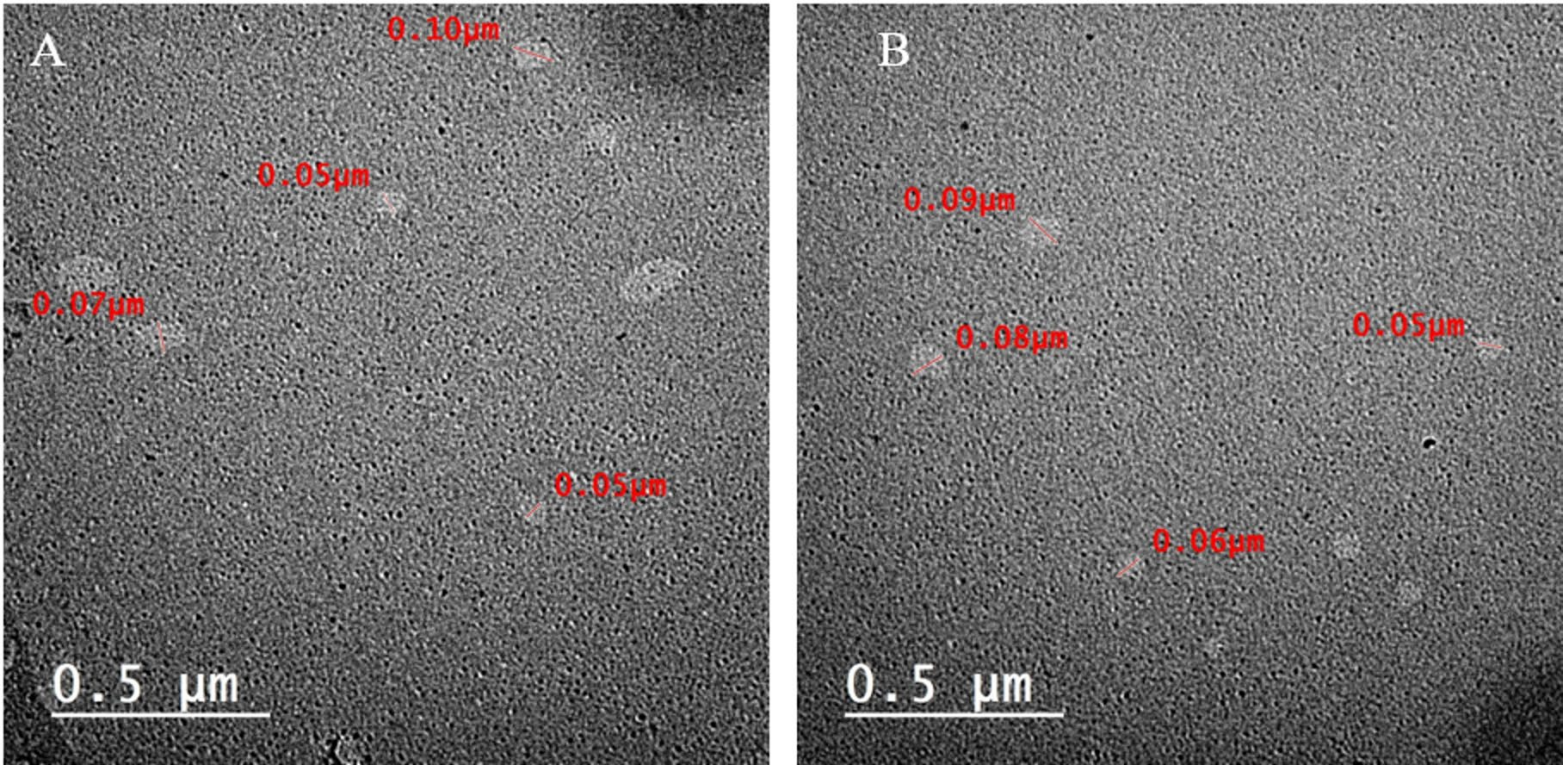
TEM of exosomes; (**A**) MSCs-Exos, (**B**) MT/MSCs-Exos, showing the characteristic spherical morphology and size range of extracellular vesicles.

Protein concentration of extracted exosomes was 1 mg/mL. The morphological characteristics of exosomes are shown in Fig. 4, which revealed that isolated exosomes exhibit a characteristic double-membrane, spheroidal vesicular structure with a diameter of 50–100 nm, as observed at a 0.5 μm scale. The observed extracellular vesicles were confirmed as exosomes by flow cytometry detection of exosomal CD markers, CD63 and CD81. Both Exos and MT/Exos showed positive expression of the measured BMMSC-Exos CD markers, and negative expression for CD45, as represented in Fig. 4S1 and Fig. 4S2.

### Efficacy of MT/MSCs‑derived exosomes in the improvement of liver function after CCl4 intoxication

CCl4 administration for nine consecutive weeks induced hepatic cellular damage and liver dysfunction. As illustrated in Table 2; Fig. 1S and S (A), CCl4-intoxicated rats showed a substantial increase in serum AST and ALT activity, accompanied by a reduction in albumin levels, compared with normal controls. However, administration of Exos and MT/Exos significantly reduced AST and ALT activity, and significantly increased serum albumin levels in the treated groups compared to the CCl4 group. Notably, MT-Exos exhibited a superior restoration of these biochemical parameters to values closely approximating those of the control group; there is no significant difference between MT/Exos and normal control at P value *<* 0.05.

**Table 2:** Effect of Exos and MT/Exos treatment on ALT, AST, albumin, and oxidant/antioxidant markers in experimental rats.

Parameters Groups	ALT U/L	AST U/L	ALB mg/dl	Nrf2 protein levels relative to β-actin	SOD pg/mg protein tissue	MDA pg/mg protein tissue
Normal	28.7 ± 1.9^c^	21.4 ± 1.3^c^	4.17 ± 0.13^a^	1.082 ± 0.07 ^d^	24.69 ± 0.39^a^	35.6 ± 2.66^c^
CCl4	281.5 ± 23.3^a^	80.1 ± 1.1^a^	2.75 ± 0.11^c^	0.089 ± 0.04 ^a^	12.70 ± 0.59^c^	82.4 ± 3.6^a^
**Exos**	**69.6 ± 2.5** ^**b**^	**34.8 ± 1.8** ^**b**^	**3.55 ± 0.11** ^**b**^	**0.648 ± 0.04** ^**b**^	**21.78 ± 0.92** ^**b**^	**60.2 ± 2.7** ^**b**^
**MT/ Exos**	**29.7 ± 1.2** ^**c**^	**26.7 ± 2.6** ^**c**^	**4.01 ± 0.16** ^**a**^	**0.852 ± 0.05** ^**c**^	**23.05 ± 0.24** ^**a, b**^	**44.3 ± 3.9** ^**c**^

Data are expressed as mean ± SE, *n* = 6, where n represents the number of rats. Different letters indicate significance at *P* < 0.05. Similar letters are not significantly different at the *P* < 0.05 level.

### MT/MSCs‑derived exosomes improved redox-sensitive signals in CCl4-induced liver fibrosis

As shown in Table 1, CCl4-intoxicated rats had a significant decrease in Nrf2 levels and SOD activity, with concomitant elevation in MDA levels, compared with normal rats. Exos and MT/Exos groups showed a marked increase in Nrf2 levels and SOD activity, accompanied by a significant reduction in MDA levels, relative to the CCl4 group. Notably, the MT/Exos-treated rats exhibited a stronger antioxidant response compared to the Exos-treated rats, indicating a superior capacity in mitigating oxidative stress. MT/Exos and the normal control group don’t have a significant difference in MDA levels at P value *<* 0.05.

### MT/MSCs‑derived exosomes reduce pro-inflammatory cytokines

**Fig. 5:**
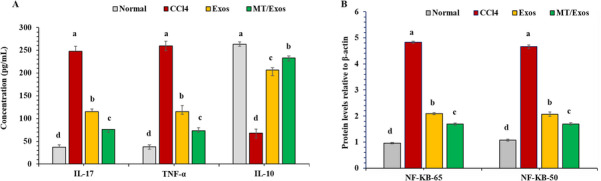
Effect of Exos and MT/Exos treatment on inflammatory markers, A includes IL-17, TNF-α, and IL-10. B, includes NF-KB P50 and p65 in experimental rats. Data are expressed as mean ± SE (*n* = 6), where n represents the number of rats. Different letters indicate significance at *P* < 0.05. Similar letters are not significantly different at P value *<* 0.05.

As shown in Figs. 1S, 2S and 5 (B and C), the CCl4 group showed a significant increase in hepatic TNF-α, IL-17, and NF-kB subunits p65 and p50, accompanied by a significant reduction in IL-10 compared with the normal control group. Following supplementation with Exos and MT/Exos, IL-10 levels were increased in the treated rats, with a significant difference compared to CCl4-intoxicated rats. Conversely, TNF-α, IL-17, and NF-κB subunits p50 and p65 levels were significantly decreased in rats treated with Exos and MT/Exos relative to the CCl4-exposed rats. A better improvement in antifibrotic capacity was observed in the MT/Exos group than in the Exos group.

### Efficacy of MT/MSCs‑derived exosomes on CCl4-triggered liver apoptosis

**Fig. 6:**
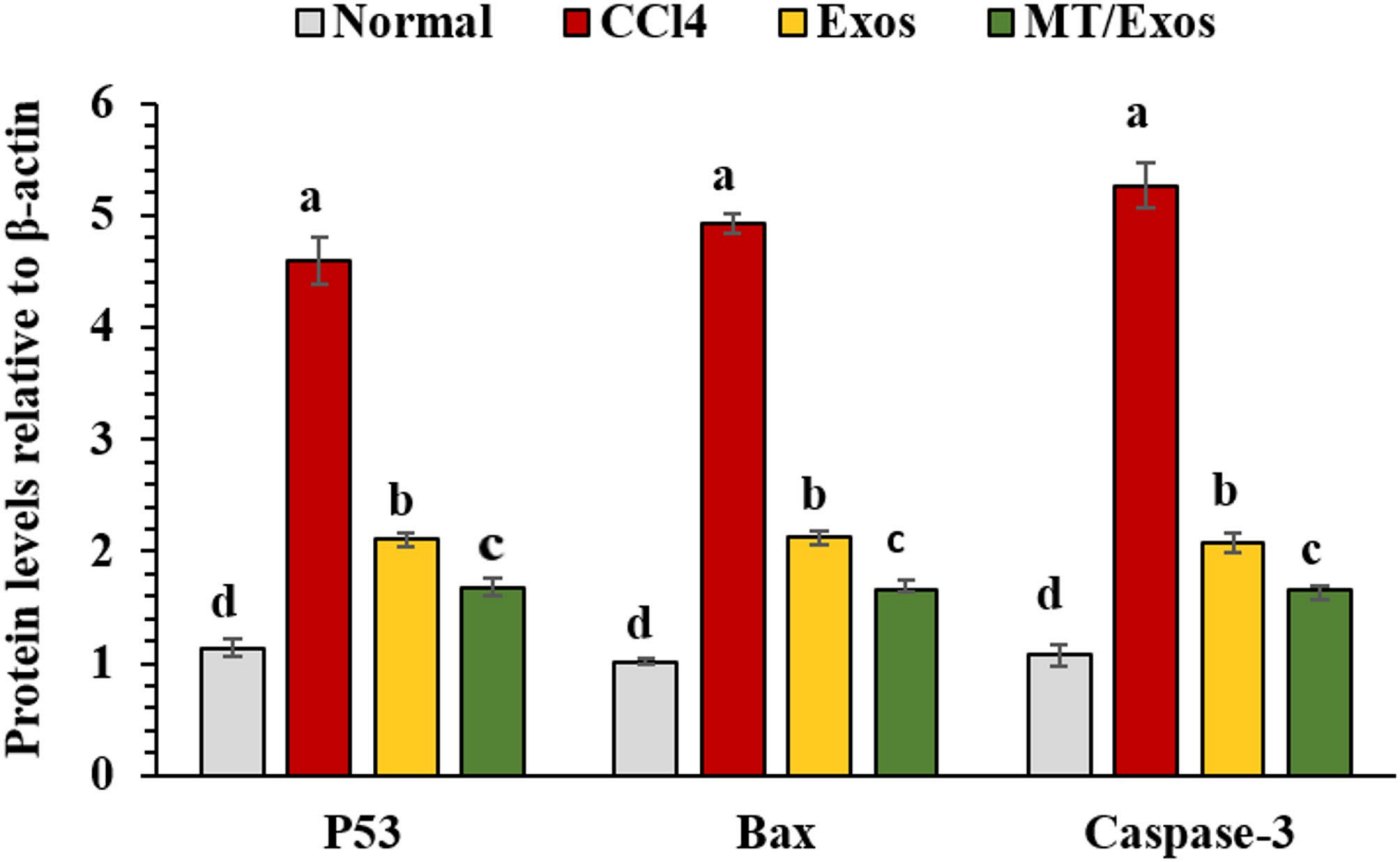
Effect of Exos and MT/Exos treatment on apoptotic markers, P53, Bax, and caspase-3 in experimental rats. Data are expressed as mean ± SE (*n* = 3), where n represents the number of rats. Different letters indicate significance at *P* < 0.05. Similar letters are not significantly different at P value *<* 0.05.

As shown in Figs. 1S, 3S and 6 (D and E), CCl4-intoxicated rats demonstrated a significant elevation in hepatic p53, Bax, and caspase-3 protein levels compared to normal control rats. Rats treated with Exos and MT/Exos showed a significant reduction in the assessed apoptotic markers compared with CCl4-intoxicatd rats. MT/Exos showed higher efficacy in reducing p53, Bax, and caspase-3 values compared with CCl4-treated rats.

### MT/MSCs‑derived exosomes suppress profibrotic signals, including TGF-β1, SMAD3, and collagen I

**Fig. 7:**
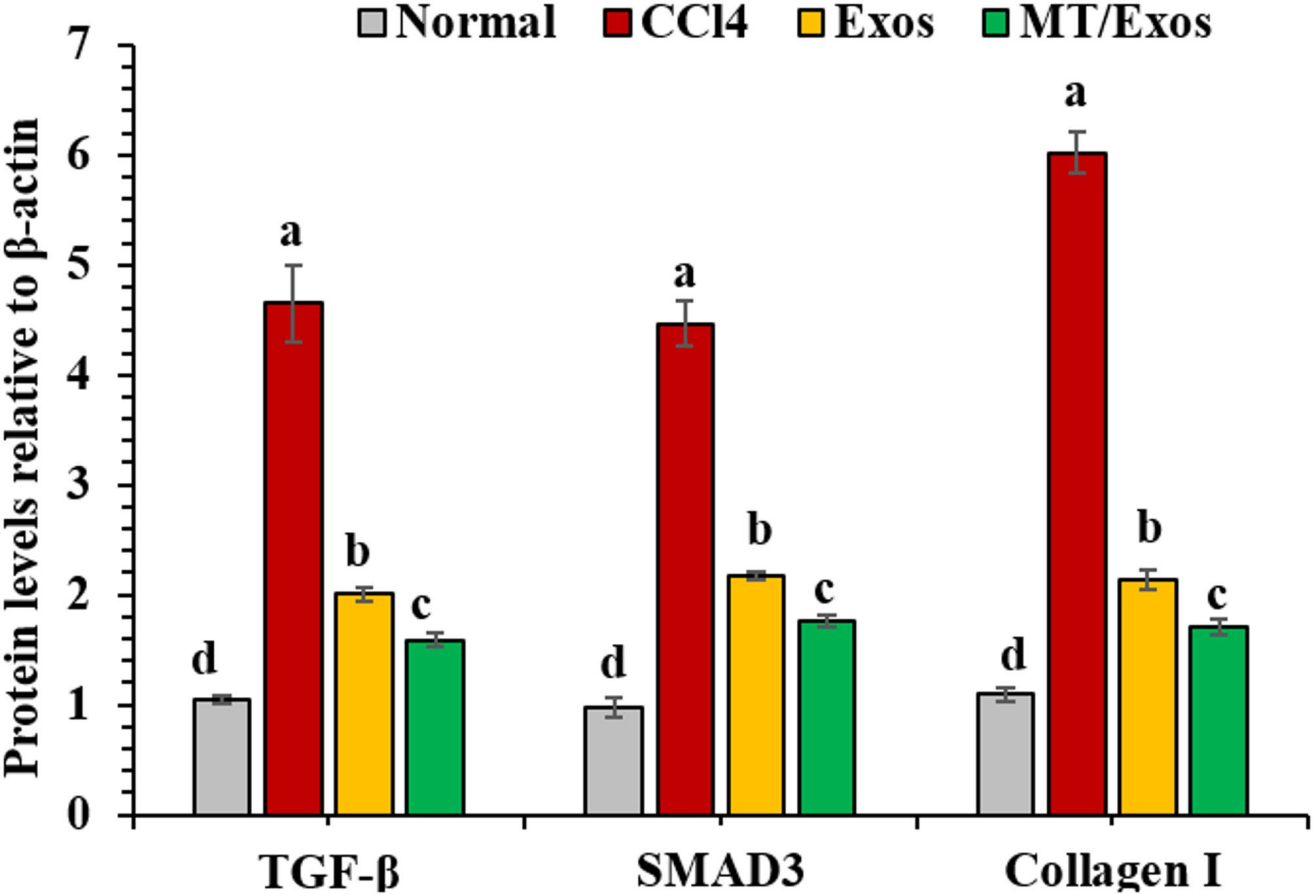
Effect of Exos and MT/Exos treatment on fibrotic markers, TGF-β, SMAD3, and collagen I in experimental rats. Data are expressed as mean ± SE (*n* = 3), where n represents the number of rats. Different letters indicate significance at *P* < 0.05. Similar letters are not significantly different at P value *<* 0.05.

As presented in Figs. 1S, 4S and 7(F-H), CCl4-intoxicated rats exhibited a marked elevation in protein levels of TGF-β, SMAD3, and collagen I compared with the normal rats. Exos and MT/ Exos groups, indicating a significant decrement in values of those estimated markers when compared to the CCl4 group. The values of the MT/Exos group are closer to those of the normal control group.

### MT/MSCs‑derived exosomes induced downregulation in miRNA-196 (miR-196) expression in treated rats

**Fig. 8:**
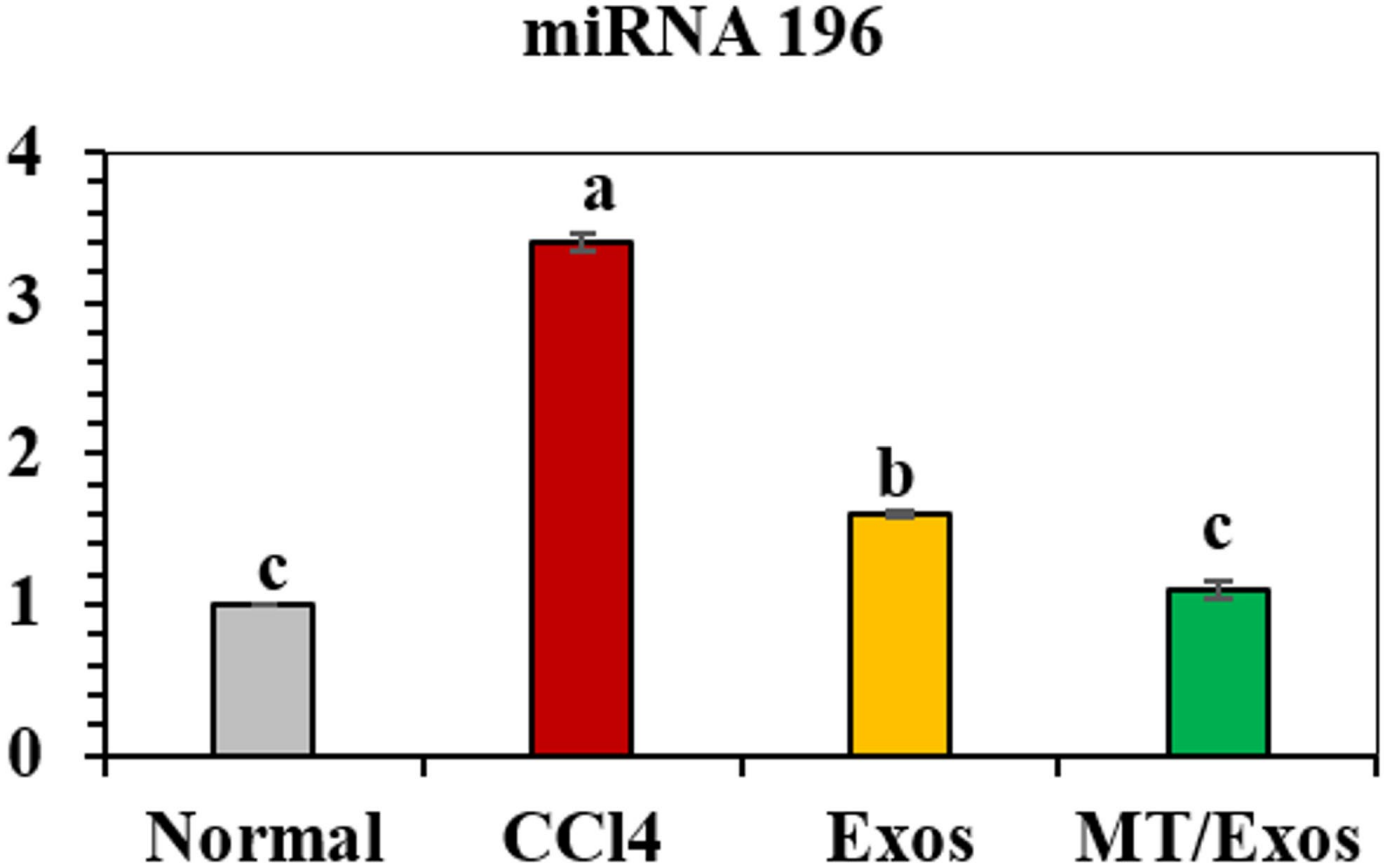
Effect of Exos and MT/Exos treatment on miRNA 196 gene expression. Data are expressed as mean ± SE (*n* = 3), where n represents the number of rats. Different letters indicate significance at *P* < 0.05.

At the molecular level, CCl4-intoxicated rats indicated a significant upregulation in miR-196 expression compared with normal rats. Meanwhile, treated groups exhibited a notable downregulation of miR-196 expression relative to the CCl4 group. MT/Exos-treated rats showed values close to those of normal control rats (Fig. 8).

### MT/MSCs‑derived exosomes reverse histological changes of liver fibrosis and improve liver cell integrity in treated rats

**Fig. 9:**
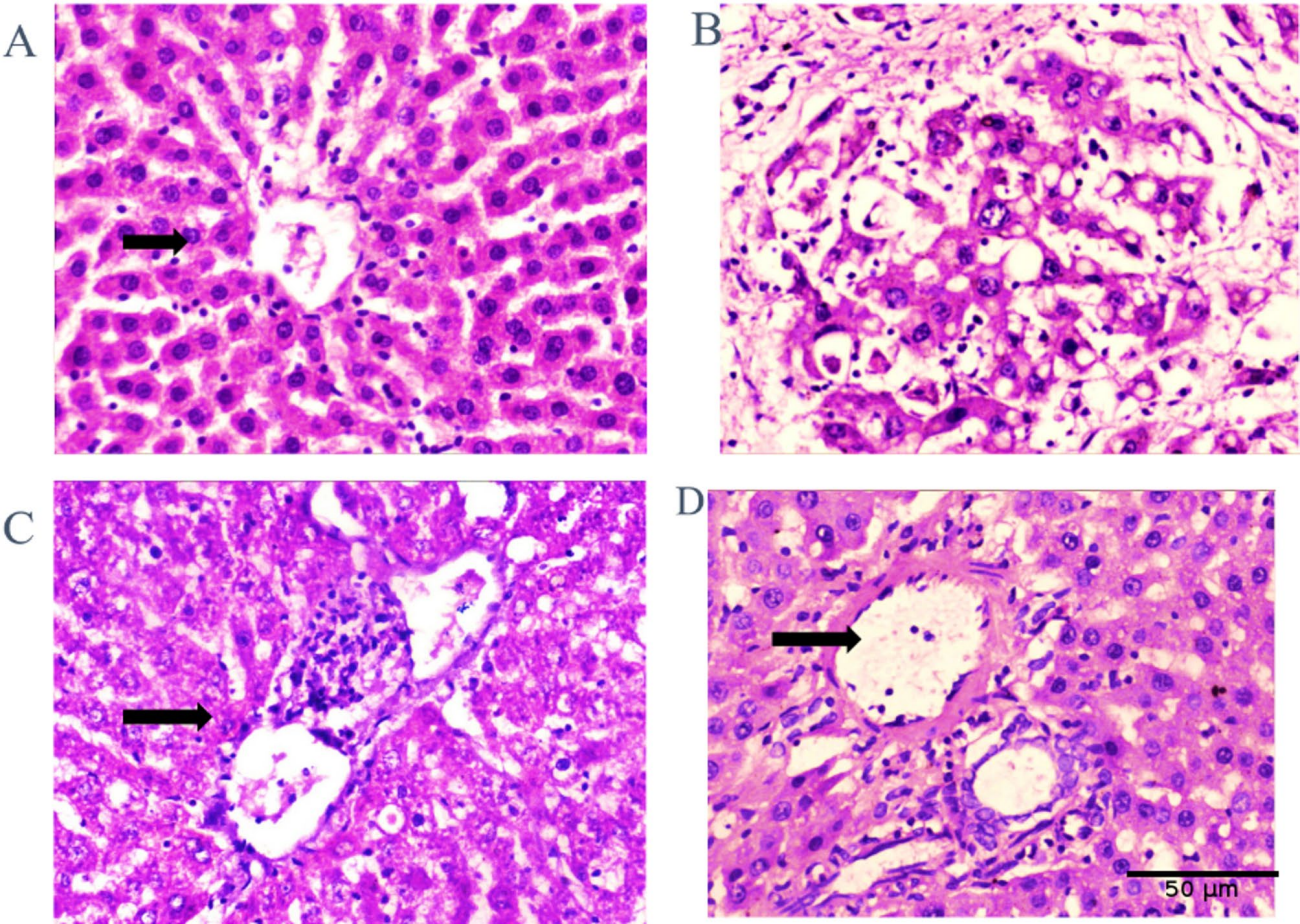
H&E stain of liver tissues 400 X; A: normal control, B: CCl4, C: Exos, and D: MT/Exos. The black arrow refers to the central vein.

**Fig. 10:**
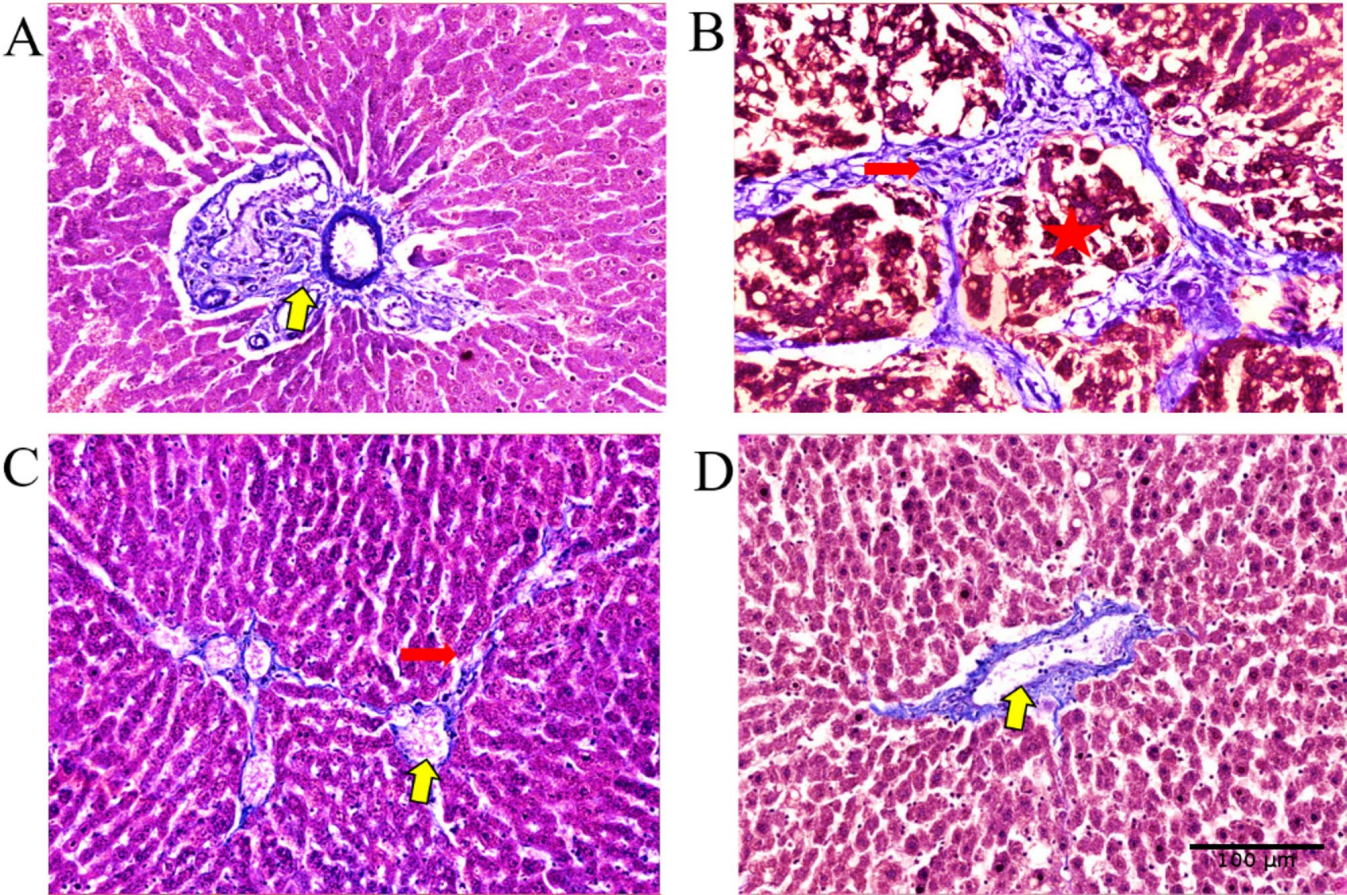
Masson trichrome stain (200 X) of liver tissues: (**A**) normal control, (**B**) CCl4, (**C**) Exos, and (**D**) MT/Exos. The yellow arrow refers to the central vein, the red arrow refers to collagen fibers, and the red star indicates regenerating nodules.

As observed in Fig. 9 (H&E stain, 400X), Fig. 10 (Masson trichome stain, 200X), and Table 3 S (METAVIR scoring system), the normal control showed a standard histoarchitecture with no signs of inflammation or fibrosis (F0). The central vein and hepatocytes appeared in normal structure. On the other hand, Fibrosis and inflammatory infiltration were visibly apparent surrounding the central vein of the injured livers in the CCl4-intoxicated rats (F4). Most hepatocytes had vacuolations and eosinophils in the cytoplasm, as well as darkly pigmented nuclei. The portal areas of the damaged liver express a significant deposition of collagen fiber, as investigated by Masson’s trichrome staining, which indicates the successful establishment of liver fibrosis in the CCl4 rats. Significantly, the Exos (F2) and MT/Exos (F1)-treated groups showed less evidence of inflammatory infiltration and liver fibrosis than the fibrosis CCl4 group, as represented in Table 3 S. Treated rats in Exos and MT/Exos groups showed improvement in liver histology, which revealed in less degeneration of hepatocyte cells and the central vein. The Exos group showed mild inflammation within the portal tract with fibrous septa formation (F2). The Masson’s trichrome results revealed less observation of collagen fiber in the treated rats’ hepatic sections. The collagen deposition in MT/Exos sections has almost disappeared compared to the CCl4 group Masson sections.

## Discussion

Liver fibrosis describes the pathological buildup of ECM in response to hepatic injury, acting as a precursor to cirrhosis and Hepatocellular carcinoma^5^. At present, no pharmacological agent approved for clinical use has demonstrated the capacity to fully reverse liver fibrosis, particularly in its advanced stages. Although stem cell therapy, specifically MSCs, has revealed promising antifibrotic effects in animal models, the expected therapeutic benefits have not been consistently achieved in most clinical trials due to cellular rejection and tumorigenicity^40^. In contrast, MSC-derived exosome-based therapy has developed as a novel therapy to overcome many limitations of MSC treatment. Furthermore, MSC preconditioning could enhance its biological activity and affect the therapeutic efficacy of its-derived exosomes. Based on this rationale, we aimed to evaluate the enhancement effect of melatonin on the anti-fibrotic potential of MT/Exos in CCl₄-induced liver fibrosis.

In our study, bone marrow was collected from male Wistar albino rats. Passage four from treated and untreated MSCs was used to isolate extracellular vesicles, mainly exosomes. MSCs were cultured and identified morphologically and by surface antigen markers. Purified MSCs exhibited positive expression of the characteristic mesenchymal CD markers CD73, CD90, and CD105, whereas CD34 is negatively expressed. The purified Exos and MT/Exos demonstrated a protein concentration of 1 mg/ml. Moreover, HR-TEM showed spherical membrane-bound nanoscale vesicles with a diameter of 50–100 nm, displaying a double-membrane-like outline. The exosomes distribution in the TEM field appeared homogeneous with no aggregations. Flow cytometry of the present data demonstrated a positive expression of CD63 and CD81. Consistent with previous studies describing MSC-exosomes, our results confirmed that the isolated vesicles were BM-MSCs-derived Exosomes^34,41,42^. Expression of CD81 and CD63, supporting the biological importance and quality of the isolated exosomes, though they play functional roles in cargo sorting and vesicle uptake^43^.

In the present study, liver dysfunction was observed as increased ALT and AST transaminases and diminished albumin levels in the serum of CCl4-intoxicated rats. Elevation of transaminases above normal values is a strong indicator of hepatocellular injury, revealing disturbed membrane integrity and leakage of cytosolic enzymes into the circulation^44^. Since hepatocytes are the only source of albumin synthesis, a decrease in serum albumin levels indicates impaired hepatocyte synthetic capacity^45^. Administration of MT/Exos induced a marked decrease in assessed transaminases and an elevation in albumin levels in treated rats. The depletion of enzyme levels indicates exosomes’ cytoprotective ability, mitigating hepatocellular membrane disruption and thereby preventing enzyme leakage into the circulation. Furthermore, elevated serum albumin in treated rats indicates restoration of liver synthetic function and recovery of protein metabolism. This outcome is in agreement with a previous report demonstrating the efficacy of exosomes in restoring liver enzymes and enhancing albumin synthesis^46^. Among the treated groups, MT/Exos revealed the closest restoration of values toward normal levels.

Oxidative stress is an essential mechanism underlying CCl4-induced hepatotoxicity. In the liver, Cytochrome P450 enzymes catalyze the biotransformation of CCl4, resulting in the production of reactive free radicals that initiate lipid peroxidation in the liver tissues^44^. This was confirmed in the present study, where we assessed MDA, SOD, and Nrf2 levels in liver homogenate. CCl4-intoxicated rats exhibited an abnormal elevation in hepatic MDA level, and a reduction in SOD and Nrf2 levels. After treatment with MT/Exos, the treated rats showed decreased MDA values and increased SOD and Nrf2 levels. MT/Exos enhanced liver antioxidant capacity, thereby inhibiting CCl4-induced oxidative stress. This result aligns with a previous study reporting that MSCs-Exos elevated Nrf2 levels in the liver^47^. In addition, the antioxidant capacity of MSCs could be particularly relevant in the case of immune and inflammatory components. Importantly, MT is involved in the detoxification of ROS and free radical intermediates because it plays a critical role in the release of antioxidant enzymes, including catalase, glutathione reductase, superoxide dismutase, and glutathione peroxidase^48^. Also, melatonin acts as a potent homeostatic and cytoprotective molecule that safeguards MSCs against oxidative stress, inflammation, apoptosis, ischemic injury, and cellular senescence, thereby contributing to the regulation of MSC differentiation and enhancing their protective functions across various tissues and organs^49^. In particular, recent intellectual property developments support the use of melatonin as a preconditioning agent to improve the biological activity and therapeutic efficacy of MSC-derived exosomes in tissue repair and fibrosis-related disorders.

Regarding the inflammatory pathway, CCl4-intoxicated rats showed elevations in hepatic TNF-α, IL-17, and NF-κB (p50 and p65), with a reduction in IL-10. In line with our data, Previous reports demonstrated that elevations in TNF-α and NF-κB are associated with liver damage and fibrogenesis^50–52^. Tiegs and Horst reported that TNF-α elevation induced hepatocyte injury and fibrogenic signaling via activation of Kupffer cell–driven inflammation^53^. A decrease in IL-10 lower than normal indicates a reduction in the liver’s intrinsic anti-inflammatory capacity^54^. Administration of MT/Exos alters all these inflammatory modulators; MT/Exos-treated rats showed a marked decrease in TNF-α, IL-17, and NF-κB, while IL-10 was markedly elevated. The depletion of TNF-α, IL-17, and NF-κB indicated that MT/Exos can downregulate the transcription of pro-inflammatory cytokines, thereby mitigating liver inflammation. In addition, IL-10 elevation by MT/Exos treatment reflects its ability to protect hepatocytes from immune-mediated injury^55,56^. All these findings confirmed the capacity of MT/Exos to rebalance the cytokine environment toward an anti-inflammatory state in the liver. Notably, MT/Exos are more effective than untreated Exos in modulating immune responses and restoring hepatic homeostasis. This observation supported the hypothesis that melatonin enhances exosome efficacy in ameliorating inflammatory response. Our findings are consistent with a recent study demonstrating that melatonin upregulates MSCs’ osteogenic action through inhibiting the NF-kB pathway^57^. Another study reported that melatonin improves the ability of MT/Exos against renal ischemia-reperfusion injury^29^.

To further confirm the efficacy of MT/Exos in suppressing hepatocyte apoptosis, the apoptotic pathway was assessed in treated and untreated rats. Our results showed that CCl4 induced higher elevations in the assessed apoptotic markers, p53, Bax, and cleaved caspase-3. This finding indicates that CCl4 supplementation for 9 weeks triggers apoptosis predominantly via the p53/Bax/caspase-3 mitochondrial pathway. Moreover, lipid peroxidation and DNA damage result from CCl4 toxicity, leading to p53, a crucial transcription factor, being overexpressed. P53 promotes transcription of Bax and other pro-apoptotic genes, which induce permeabilization of the mitochondrial membrane and trigger caspase activation, such as caspase-3. Furthermore, caspases mediate the terminal stages of apoptosis^58,59^. The protective role of MT/Exos on hepatocyte survival was demonstrated by decreased p53, Bax, and cleaved caspase-3 values in MT/Exos-exposed rats. This suggests the efficacy of MT/Exos in inhibiting apoptosis and enhancing overall hepatoprotection. In agreement with our study, a previous report demonstrated the efficacy of exosomes in suppressing apoptosis in a pig model of liver injury^60^. The present study confirmed the efficacy of MT/Exos to protect liver tissues from inflammation, lipid peroxidation, and apoptosis. To clarify the anti-fibrotic mechanisms following MT/Exos administration, we investigate levels of key mediators of liver fibrosis that could directly affect HSC activation, a hallmark of progressive fibrosis. The measured parameters include TGF-β, SMAD3, and collagen I. It was reported that TGF-β/SMAD3 signaling stimulates HSCs activation and ECM deposition^61^. Activated HSCs stimulate collagen I production, enhance ECM buildup, and disrupt the normal architecture of the liver^37^. In our data, CCl4-intoxicated rats showed a significant elevation in all evaluated fibrotic modulators compared with rats in the normal and treated groups. After Exos and MT/Exos treatment, a marked decrease in TGF-β and SMAD3 fibrotic markers was observed, and collagen I protein levels were depleted. The promising reduction of TGF-β and SMAD3 in the MT/Exos group indicates attenuation of this central pathway, which mechanistically clarifies the observed decline in collagen deposition revealed in histology images. Collectively, the previous finding confirmed that MT/Exos treatment could reverse liver fibrosis by decreasing TGF-β levels and inhibiting HSC activation, thereby suppressing collagen I release and improving liver histology. Along with our results, recent work illustrated that Wartton jelly -MSC exosomes can directly inhibit the TGF-β/SMAD3 pathway, therapy suppresses HSC activation and mitigates fibrosis^62^. Taken together, these recent studies indicate that liver fibrosis is closely driven by oxidative stress, inflammatory responses, and activation of fibrogenic pathways^63^. In contrast, therapeutic strategies based on stem cell–derived exosomes, antioxidants, and nano-regenerative approaches have been shown to counteract these mechanisms, thereby reducing fibrosis and supporting liver tissue recovery.

In addition, it has been reported that the use of melatonin as a preconditioning agent could enhance the bioactive cargo of MSC-derived exosomes, enriching them with antioxidant and anti-inflammatory molecules such as specific microRNAs and proteins^64^. Molecular investigations in our study confirmed a significant upregulation of miR-196 expression in CCl4-intoxicated rats compared with normal rats. In this line, miRNAs have been implicated in fibrogenesis and liver injury pathways. A recent study showed that miR-196 regulates collagen I expression and the TGF-β signaling pathway^65^. miR-196 is reported to regulate genes involved in ECM remodelling and inflammatory responses in liver injury^66^. Meanwhile, the administration of Exos and MT/Exos induced a marked downregulation of miR-196, as revealed in treated rats relative to CCl4-intoxicated rats. Serum miR-196 downregulation is associated with improvements in biochemical markers and liver tissue architecture in treated rats. Changes in serum miR-196 levels between treated and untreated rats are evidence for the molecular mechanisms mediating exosomes’ anti-fibrotic capability. In agreement with this finding, Rui et al. demonstrated that exosomal miRNAs, including members of the miR-196 family, can serve as both biomarkers and effectors in liver disease^67^. Accordingly, additional investigations using miR-196 inhibition strategies are warranted to further confirm its functional contribution to the observed antifibrotic effects and to better elucidate the underlying molecular mechanisms involved. Meanwhile, future studies should include both sexes to evaluate potential sex-related differences in response to MT/Exos treatment. The promising results with MT/Exos in this preclinical model suggest potential for translation to human liver fibrosis. However, several challenges remain, including: (1) scalable production of clinical-grade exosomes, (2) standardization of melatonin preconditioning protocols, (3) determination of optimal dosing regimens in humans, and (4) assessment of long-term safety. Despite these challenges, exosome-based therapies offer advantages over cell-based approaches, including reduced immunogenicity and the ability to be stored as off-the-shelf products. Moreover, further studies using relevant human hepatic cell lines and human-derived stellate cell models are warranted to better clarify the molecular mechanisms underlying MT/Exos activity and to support the clinical translation of this therapeutic approach.

Histopathological evaluations in our study confirmed well-established liver fibrosis and revealed the efficacy of Exos and MT/Exos in improving and normalizing liver architecture, as evidenced by histological images. Pronounced collagen deposition, portal fibrosis, and inflammatory cell infiltration are clearly observed in CCl4-intoxicated rats; these lesions are associated with hepatocyte dysfunction and the HSC activation cascade. As we discussed previously, the CCl4 group showed elevations in liver enzymes, oxidative stress, inflammation, apoptosis, and profibrotic modulators, triggering HSC activation and collagen overproduction. These observations align with the standard pathology of CCl4-induced liver injury^35,68^. Masson’s trichrome staining revealed prominent deposition of collagen fiber, confirming the elevation in collagen I levels revealed in western blotting of the CCl4 group. On the other hand, treatment with Exos and MT/Exos markedly reduced both inflammatory infiltration and collagen deposition compared to the untreated fibrosis group. MT/Exo treatments reduce fibrotic scar formation in the liver tissues and restore architecture to near-normal levels. Therapy supports the biochemical and molecular benefits revealed in the treated rats. Overall, the histological data confirm that MT/Exos therapy is superior at mitigating hepatic injury and fibrosis progression in the CCl4 model.

## Conclusion

A well-isolated and characterized MT/Exos demonstrated superior ability to improve liver fibrosis. MT/Exos ameliorated the altered biochemical liver markers, restored antioxidant activities of the liver by elevating Nrf2 and SOD levels, and reducing MDA levels. MT/Exos modulate inflammation by elevating anti-fibrotic IL-10 and depleting pro-inflammatory cytokines. Moreover, MT/Exos acts as an anti-apoptotic agent by reducing p53, Bax, and cleaved caspase-3. Furthermore, MT/Exos attenuate the fibrogenic pathway by markedly decreasing TGF-β, SMAD3, miR196, and collagen I. Histopathological evaluations confirmed MT/Exos’s ability to improve hepatic architecture and reduce collagen deposition. Finally, melatonin could enhance the efficacy of MT/Exos as a novel strategy for reversing liver fibrosis. Further investigations are necessary to confirm these findings in diverse models, using human hepatic and fibrotic cells to elucidate the underlying mechanisms, and assess long-term safety and clinical applicability.

## Supplementary Information

Below is the link to the electronic supplementary material.

Supplementary Material 1

Supplementary Material 2

## Data Availability

The datasets used and/or analysed during the current study are available from the corresponding author on reasonable request.
