# Supplementary figures and images for: Melatonin-treated bone marrow mesenchymal stem cell-derived exosomes reverse liver fibrosis induced by CCl4 in male wistar albino rats
Source: Sci Rep. 2026 Jun 21;16:19195. doi: 10.1038/s41598-026-58433-x (PMC13284369; doi:10.1038/s41598-026-58433-x)

**Normal      CCl4      Exos      MT/Exos**

**Nrf2** 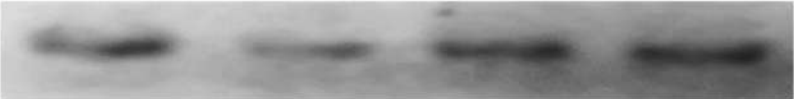 **60 KDa**

**β actin** 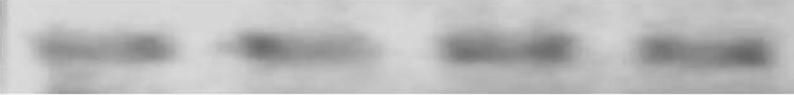 **43 KDa**

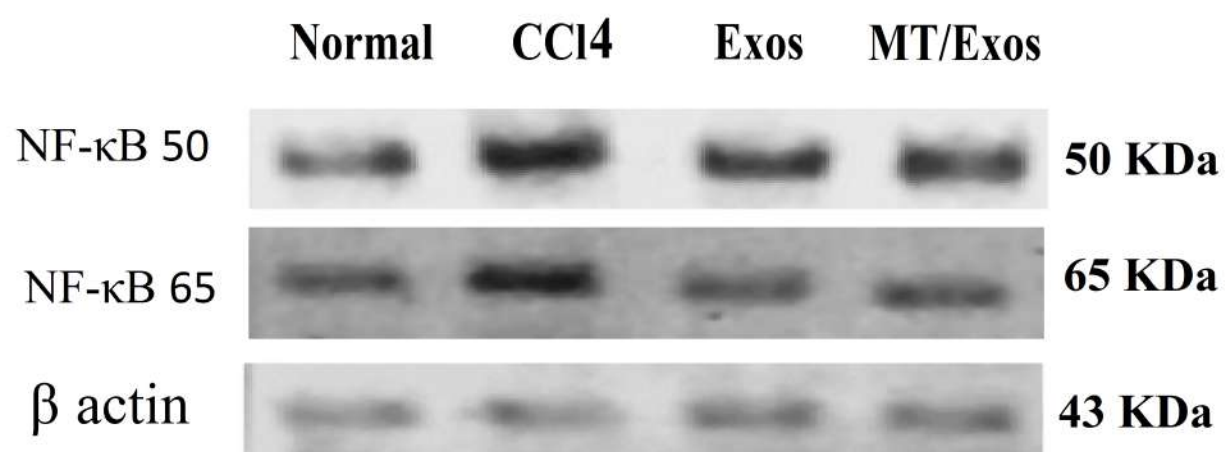

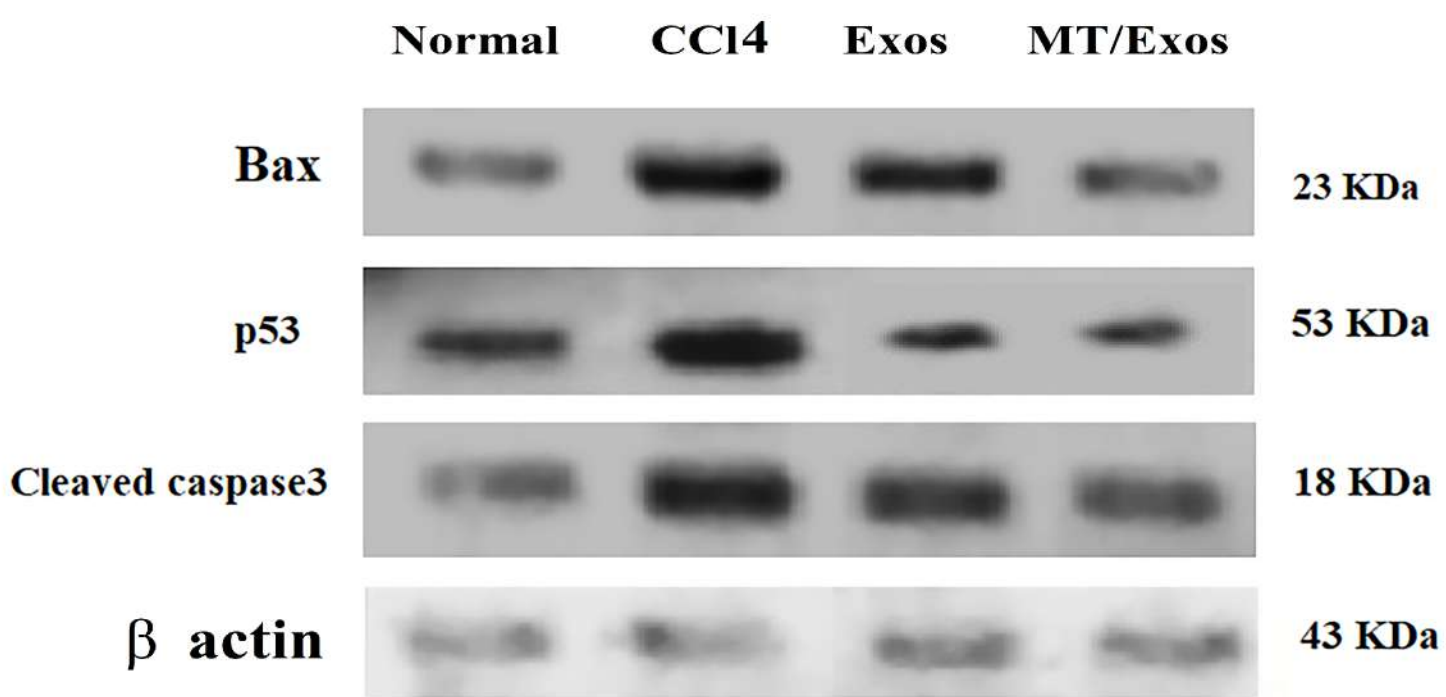

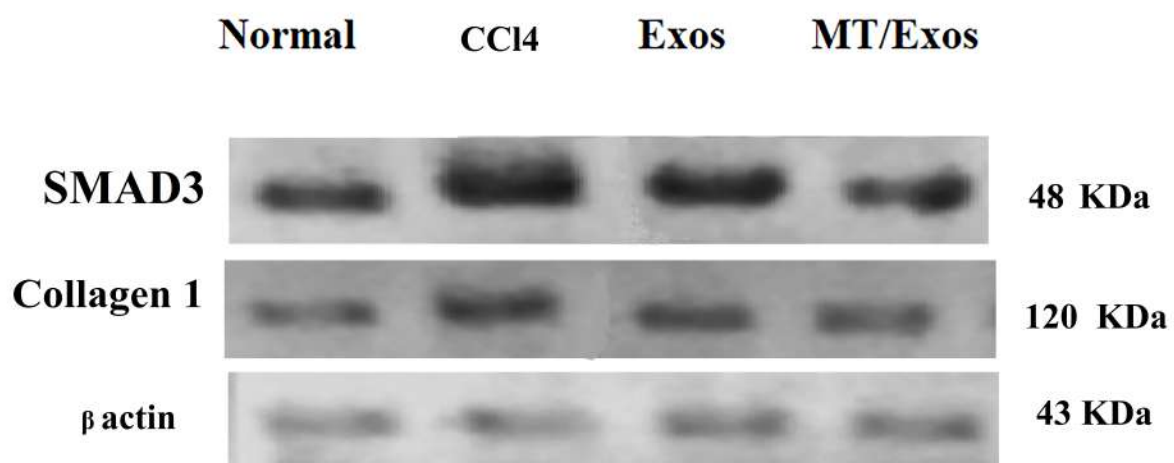

Supplement: Supplementary file 1 — Supplementary Material 1 [file 41598_2026_58433_MOESM1_ESM.pdf]
