# Supplementary material for: Melatonin-treated bone marrow mesenchymal stem cell-derived exosomes reverse liver fibrosis induced by CCl4 in male wistar albino rats
Source: Sci Rep. 2026 Jun 21;16:19195. doi: 10.1038/s41598-026-58433-x (PMC13284369; doi:10.1038/s41598-026-58433-x)
Supplement: Supplementary file 2 — Supplementary Material 2 [file 41598_2026_58433_MOESM2_ESM.docx]

Supplementary Fig. 1S(A-H): Uncropped full-length western blot data.


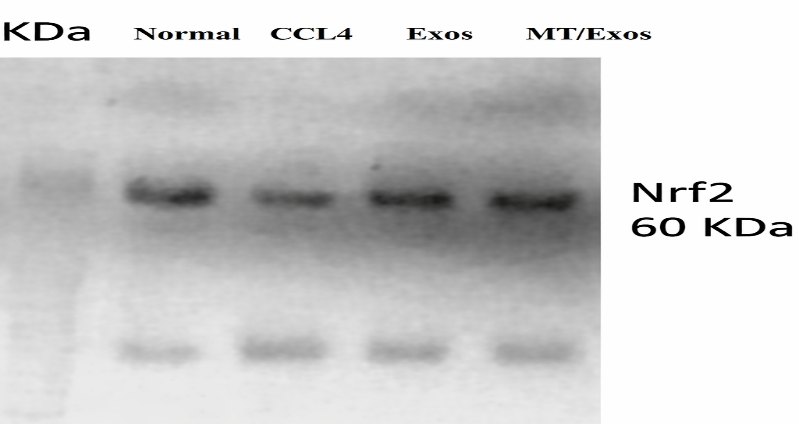


Fig. 1S(A): The uncropped data for NRF2 are as follows: normal control, CCl4, Exos, and MT/Exos


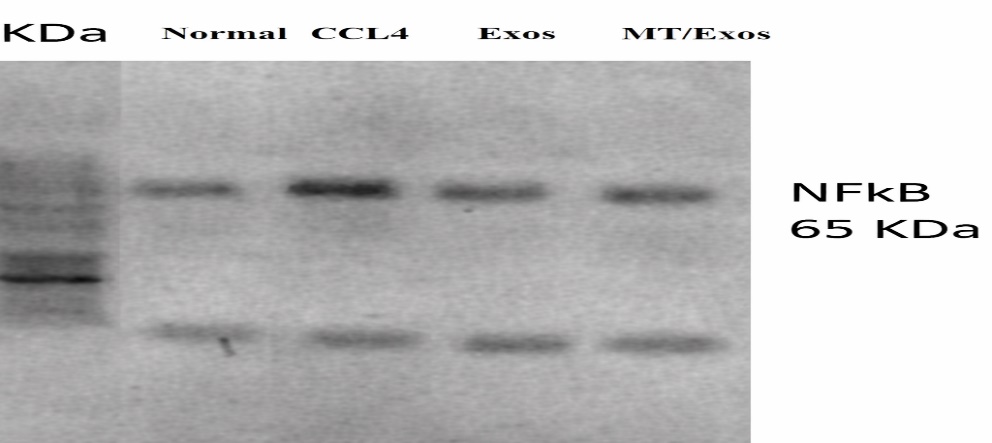


Fig. 1S (B). The uncropped data for NF-kB 65 are as follows: normal control, CCl4, Exos, and MT/Exos.


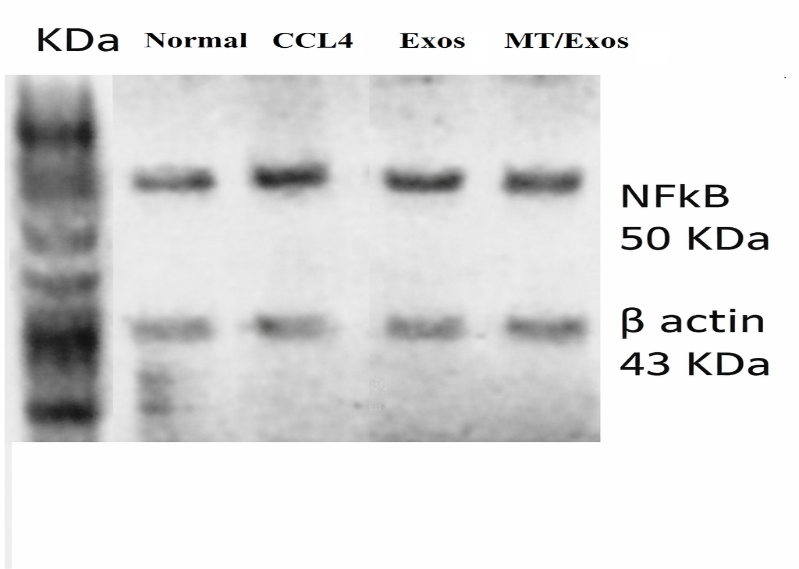


Fig. 1S (C). The uncropped data for NF-kB 50 are as follows: normal control, CCl4, Exos, and MT/Exos.


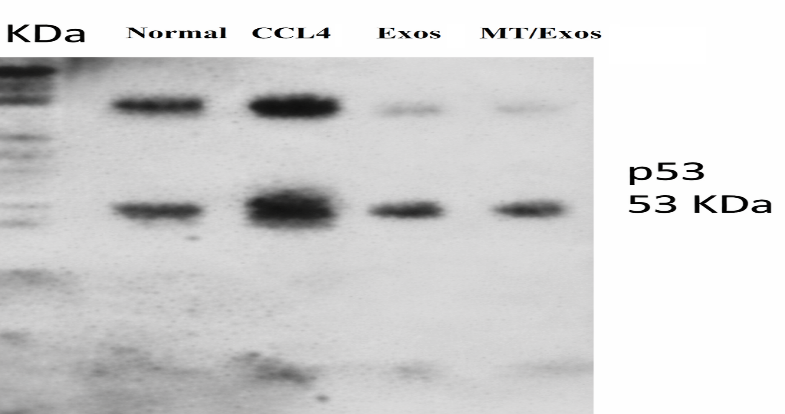


Fig. 1S(D). The uncropped data for P53 are as follows: normal control, CCl4, Exos, and MT/Exos


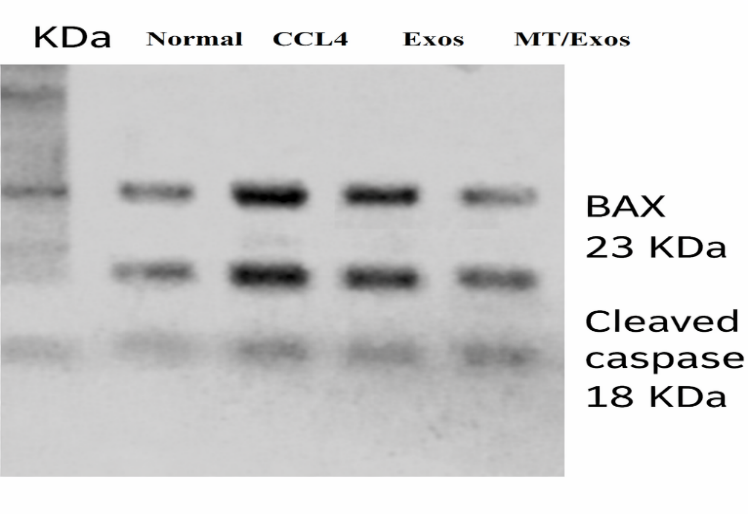


Fig. 1S (E). The uncropped data for Bax, and caspase-3 are as follows: normal control, CCl4, Exos, and MT/Exos.


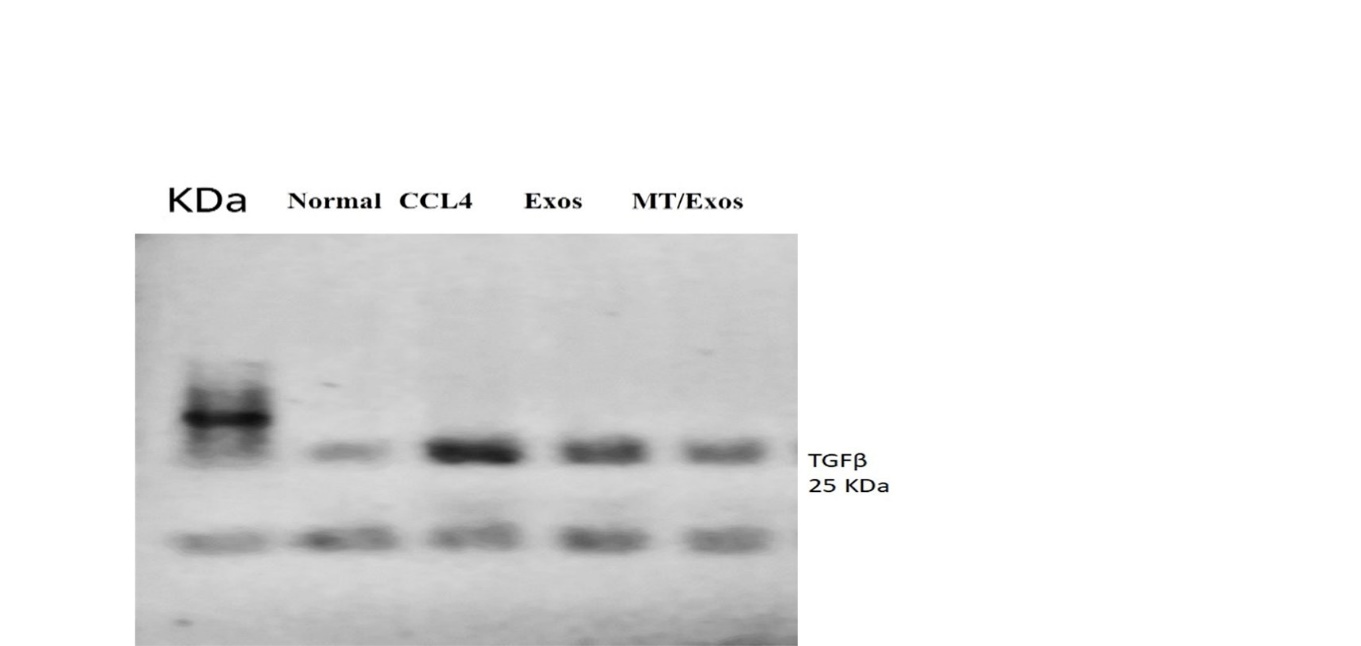


Fig. 1S (F). The uncropped data for TGF-β1 are as follows: normal control, CCl4, Exos, and MT/Exos.


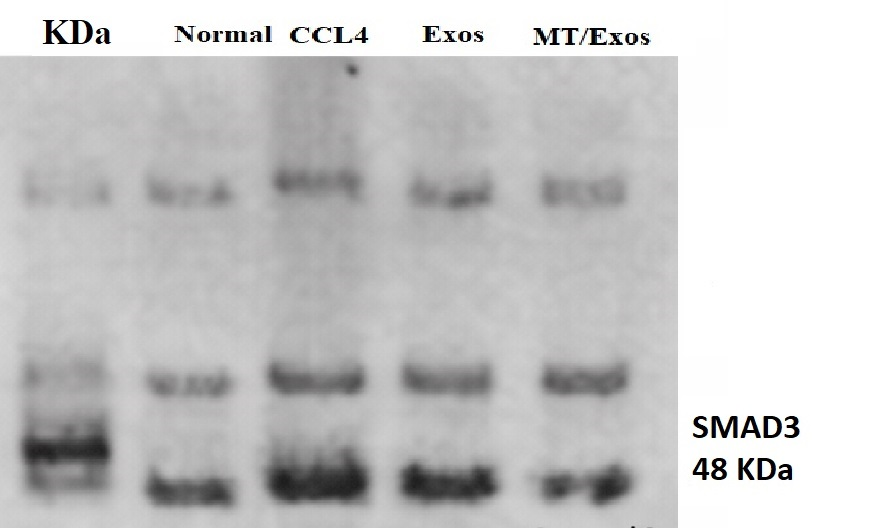


Fig. 1S (G). The uncropped data for SMAD3 are as follows: normal control, CCl4, Exos, and MT/Exos


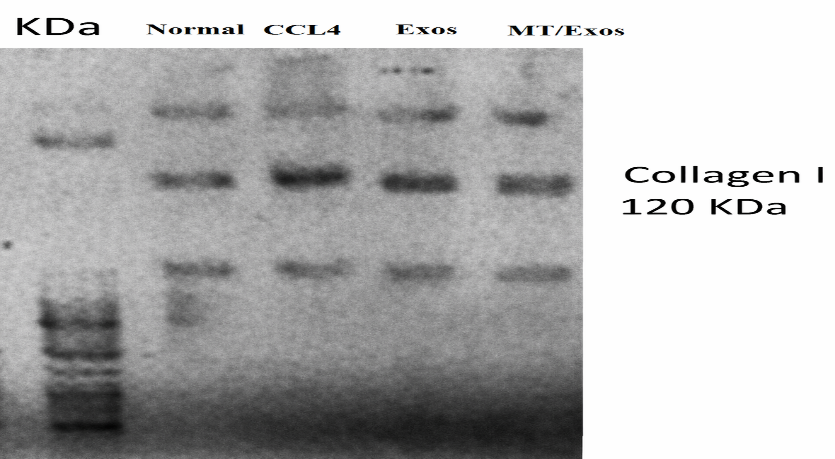


Fig. 1S (H). The uncropped data for collagen I are as follows: normal control, CCl4, Exos, and MT/Exos.


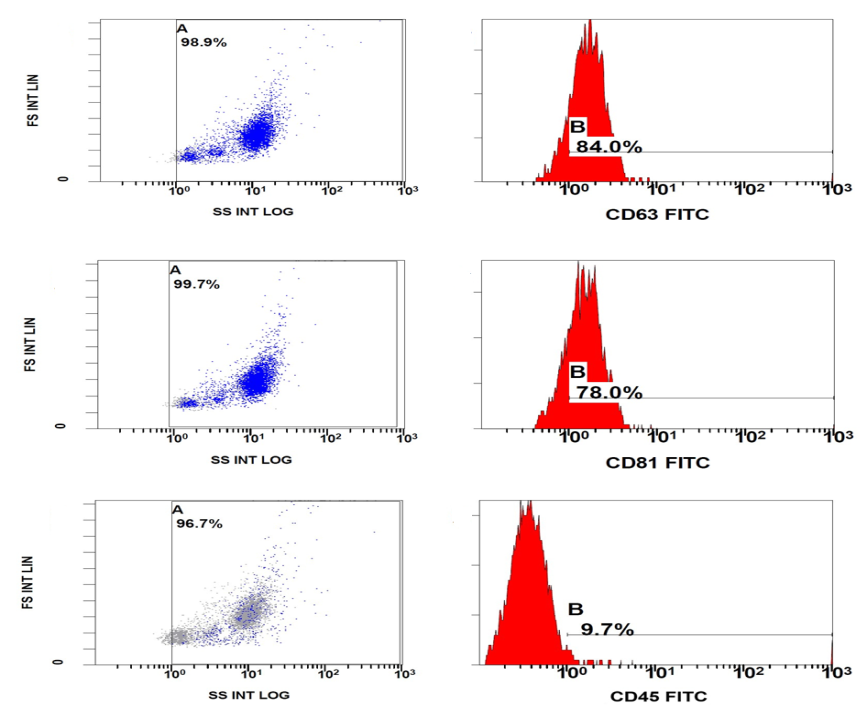


**Fig.4S1.** Flow cytometry of Exos by logarithmic scale using forward scatter (FS) and side scatter (ss); CD 63 showing positive expression by 84.0 %, CD 81 represents positive expression by 78.0%, and CD represents negative expression by 9.7 %.


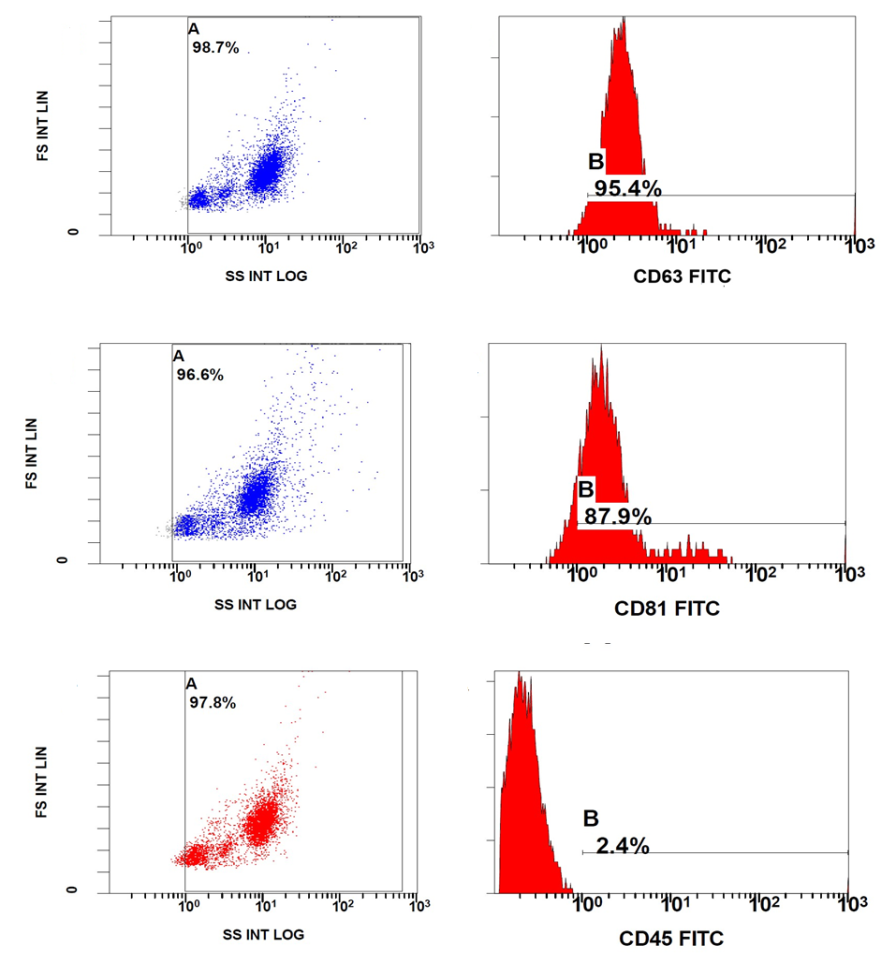


**Fig. 4S2.** Flow cytometry of MT/Exos by logarithmic scale using forward scatter (FS) and side scatter(ss); CD 63 showing positive expression by 95.4 %, CD 81 represents positive expression by 87.9%, and CD represents negative expression by 2.4 %.

**Table 3S.** **METAVIR Scoring of Liver Fibrosis in Different Experimental Groups**

| Group | METAVIR scoring | Scoring description |
| --- | --- | --- |
| Normal | **F0** | **No fibrosis** |
| CCl4 | **F4** | **Bridging fibrosis (cirrhosis)** |
| Exos | **F2** | **Portal fibrosis with fibrous septa** |
| MT/ Exos | **F1** | **Portal fibrosis without fibrous septa** |

The table summarizes the METAVIR fibrosis scores for each experimental group. The scoring system ranges from F0 to F4, indicating the severity of liver fibrosis. The fibrosis stage was scored on a scale from F0 to F4 as follows: F0, no fibrosis; F1, portal fibrosis without septa; F2, portal fibrosis with rare septa; F3, numerous septa without cirrhosis; and F4, cirrhosis

**List of abbreviations**

ALB: albumin

ALT: Alanine Aminotransferase

AST (need to be added): Aspartate Aminotransferase

BCA: Bicinchoninic Acid

CCl4: Carbon tetrachloride

CD: Cluster of Differentiation

Ct: Cycle Threshold

ECM: Extracellular Matrix

EVs: Extracellular Vesicles

Exos: Bone marrow mesenchymal stem cell-derived exosomes

fluorescein isothiocyanate: (FITC)

H&E: Hematoxylin and Eosin

HRP: Horseradish Peroxidase

HR-TEM: High-Resolution Transmission Electron Microscope

HSCs: Hepatic Stellate Cells

IL-10: interleukin-10

IL-17: Interleukin-17

MDA: Malondialdehyde

miR-196: miRNA 196

MSCs: Mesenchymal Stem Cells

MT/Exos: Melatonin-pretreated bone marrow mesenchymal stem cell-derived exosomes

MT: Melatonin

NF-κB: Nuclear Factor Kappa B

SOD: superoxide dismutase

TGF-β: Transforming Growth Factor Beta

TNFα: Tumor Necrosis Factor
